# Desmoid-type fibromatosis in the setting of malignant peritoneal mesothelioma: a case report of two rare diseases

**DOI:** 10.1186/s12957-022-02784-y

**Published:** 2022-09-28

**Authors:** Stephanie N. Gregory, Audra A. Satterwhite, H. Richard Alexander, Andrew M. Blakely

**Affiliations:** 1 Department of Surgical Oncology, National Cancer Institute, National Institutes of Health, 10 Center Drive, Bethesda, MD 20892 USA; 2 Department of Surgical Oncology, Rutgers Cancer Institute of New Jersey, Rutgers Robert Wood Johnson University Hospital, 195 Little Albany St, New Brunswick, NJ 08901 USA

**Keywords:** Malignant peritoneal mesothelioma, Mesothelioma, Cytoreduction, Hyperthermic intraperitoneal chemotherapy, Desmoid-type fibromatosis

## Abstract

**Background:**

Desmoid-type fibromatosis (DTF) is a rare benign lesion that usually arises from the abdominal wall or extremities and rarely from the mesentery or intrabdominal organs. Malignant peritoneal mesothelioma is also a rare, yet aggressive disease. To our knowledge, this is the first case report of desmoid-type fibromatosis in the setting of malignant peritoneal mesothelioma.

**Case presentation:**

An early 30-year-old female was referred to our center for large intra-abdominal mass concerning for recurrent malignant peritoneal mesothelioma after previous cytoreductive surgery with hyperthermic intraperitoneal chemotherapy and adjuvant chemotherapy. Further investigation revealed a large mesenteric mass, which was resected en bloc with the cecum and terminal ileum. Pathologic findings confirmed a surprising diagnosis of desmoid-type fibromatosis.

**Conclusions:**

No adjuvant therapy was offered to this patient due to negative tumor margins; however, close follow-up will be provided for recurrence of both malignant peritoneal mesothelioma and desmoid-type fibromatosis, which can be differentiated in the future via biopsy in this patient.

## Background

Malignant peritoneal mesothelioma and desmoid-type fibromatosis (DTF) are rare neoplasms. Malignant peritoneal mesothelioma is an aggressive and lethal disease with overall survival of 6 months at best if left untreated [1, 2]. On the other hand, DTF is a benign, yet occasionally locally aggressive neoplasm [3]. The incidence of DTF is approximately 5–6 cases per million, with 900 new cases diagnosed annually in the USA [4–6]. DTFs commonly occur in patients aged 25–35 years and twice as often in females compared to males [5]. DTFs can occur sporadically or in conjunction with hereditary syndromes such as familial adenomatous polyposis (FAP) manifesting as Gardner syndrome [7]. Other risk factors include abdominal surgery/trauma and women of childbearing age due to the influence of estrogen on desmoid tumor growth [8]. DTFs can occur anywhere in the body, most commonly in the abdominal wall and extremities, and rarely from the abdominal organs or mesentery [9]. While standard of care for resectable malignant peritoneal mesothelioma is cytoreduction and heated intraperitoneal chemotherapy (HIPEC), treatment for DTF ranges from observation to surgery. Herein, we report a rare case of desmoid-type fibromatosis arising from the mesentery in a previously diagnosed and treated patient with malignant peritoneal mesothelioma. To our knowledge, there are no case reports demonstrating this clinical scenario.

## Case presentation

**Fig. 1:**
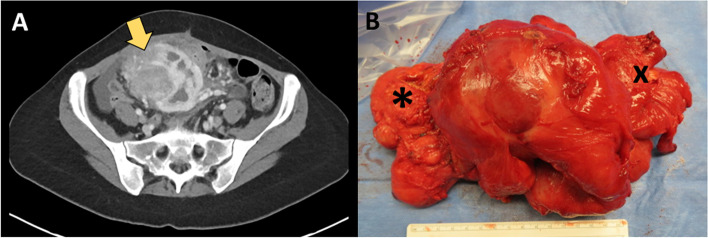
**A** Computed tomography image of intrabdominal mass (yellow arrow) and **B** en bloc resection of mass in relation to cecum (*) and small bowel (x)

A Hispanic female in her early 30s initially presented in 2017 with months of abdominal discomfort and distention. CT imaging showed a left ovarian lesion and marked ascites. Patient underwent a paracentesis and diagnostic laparoscopy which revealed peritoneal nodular implants with pathology confirming peritoneal mesothelioma with positivity for CK5/6, CK7, keratin AE3/AE1, and podoplanin (D2-40). Patient was referred to the USA in 2018 for treatment where she underwent a cytoreductive surgery with HIPEC with mitomycin C for well-differentiated epithelioid mesothelioma. Patient’s peritoneal cancer index (PCI) was 24, and completeness of cytoreduction score was 0. Tumor was positive for calretinin, CK5/6, and CAM5.2 and negative for MOC-31 and CEA. She was without symptoms or radiographic evidence of recurrence for 2 years, until she presented to her home hospital with fevers, hypotension, abdominal pain, and distention. On examination, she had a palpable abdominal mass with MRI showing a hypointense lesion with air fluid levels measuring 10.5 × 10.7 cm and multiple mesenteric nodules. She was taken for exploratory laparotomy, partial mesenteric mass excision, and evacuation of intra-abdominal abscess. Complete excision was not performed during initial operation due to involvement of surrounding bowel and mesenteric vessels. Immunohistochemistry was positive for podoplanin, calretinin, and CK5/6 and negative for desmin and anti-CD117. Patient was referred to our facility for further treatment, with restaging laparoscopy performed in December 2020. Due to prior incomplete resection and to potentially facilitate re-resection, five cycles of neoadjuvant cisplatin and pemetrexed were then administered. In June 2021, the patient underwent restaging CT which revealed essentially stability of the mesenteric mass (9.3 × 5.7 × 7.3 cm) with central air and fluid and multiple enhancing mesenteric nodules (Fig. 1A). Patient was taken to the operating room in July 2021 for mesenteric mass excision with en bloc ileocecectomy and extended terminal ileectomy (Fig. 1B).

Although additional smaller visceral peritoneal nodules (< 2.5 mm) were encountered when evaluating the bowel, repeat HIPEC was not performed due to substantial sclerosis associated with the deeper mesenteric nodules. Patient’s PCI was 2, and completeness of cytoreduction score was 0. Pathology revealed spindle cell proliferation in a collagenous stroma with nuclear staining positive for beta-catenin, positive calretinin and CK7, and negative DOG-1 staining. This pathology revealed a new diagnosis: desmoid-type fibromatosis.

## Discussion

Despite concern for recurrent peritoneal mesothelioma, this patient’s pathology revealed a benign intra-abdominal tumor: desmoid-type fibromatosis. Although DTF is not malignant, it can be a locally aggressive neoplasm that causes significant morbidity and occasional mortality [3]. While DTFs can occur anywhere in the body, they most commonly arise from the abdominal wall and extremities and rarely from the abdominal organs or mesentery [9]. In most intra-abdominal cases, patients are usually asymptomatic; however, intestinal obstruction, abscess formation, and perforation can occur if DTFs enlarge, thus requiring surgery [10].

**Fig. 2:**
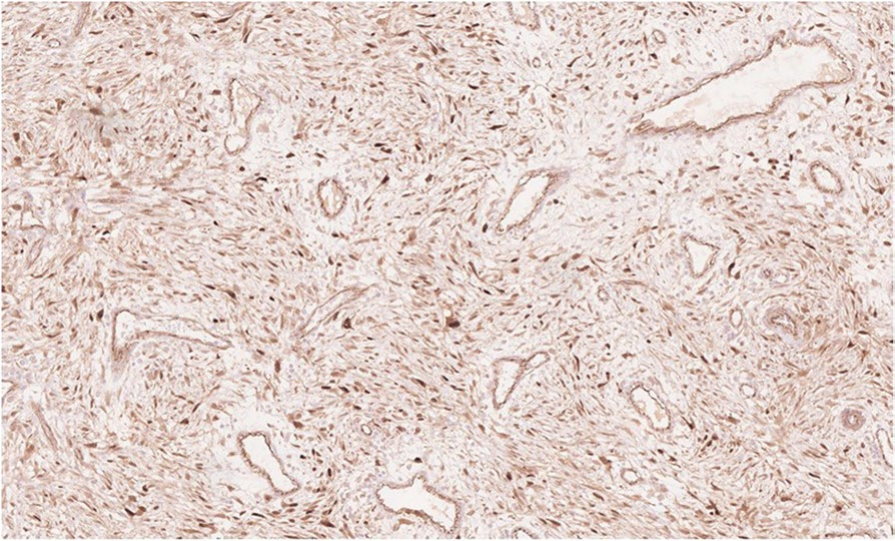
Beta-catenin nuclear positivity in spindle cells of desmoid tissue

Interestingly, there are case reports of desmoid fibromatosis diagnosed initially as malignant mesothelioma, based on immunohistochemical (IHC) calretinin positivity. Calretinin is an intracellular calcium-binding EF-hand protein that is widely used as a positive marker in diagnosis of malignant mesothelioma [11]. The most sensitive IHC markers for mesothelioma include calretinin, cytokeratin 5/6, and Wilms’ tumor (WT-1) [1, 12]. Additional markers used for epithelioid mesothelioma diagnosis include positive podoplanin (D2-40) and negative MOC-31, which were also noted in the patient’s pathology [13]. One study from the National Cancer Institute in Bethesda, Maryland, evaluated the reactivity of several IHC markers, calretinin, keratin cocktail (AE1/AE3), and WT-1 immunoreactivity to differentiate malignant mesothelioma from other fibroblastic/myofibroblastic neoplasms. Like mesothelioma, calretinin positivity was observed in 75% of desmoid fibromatosis. In contrast, DTFs were negative for keratin (AE1/AE3) and WT-1 [11]. Histologically, desmoid tissue is also positive for nuclear beta-catenin, intermediate vimentin, and COX-2 and will appear as intertwining bundles of spindle cells within a collagen matrix (Fig. 2) [14, 15].

Treatment strategies for DTF are controversial. Historically, surgical resection with microscopic negative margins was standard of care. However, a new strategy of observation has emerged due to conflicting data on the impact of complete microscopic resection on recurrence [5]. In fact, some patients who receive no treatment have shown no tumor progression and occasionally spontaneous regression [5]. This patient, however, had positive macroscopic margins after incomplete resection of the mesenteric mass during her emergency surgery and had progression of the primary tumor. A second surgery was performed for complete resection due to continued abdominal pain, tumor progression despite chemotherapy, location of tumor, and patient’s young age.

As a result of her most recent surgery, tumor margins were negative; therefore, no adjuvant therapy was offered following surgical resection. In patients who are not surgical candidates or who undergo incomplete resection, adjuvant therapy can be considered in the form of radiation or systemic therapy. Radiation may confer some benefit in terms of improved local control after incomplete resection; however, no prospective randomized data is available [5]. For intra-abdominal DTFs, the relative benefit of radiation therapy must be weighed against the high risk of radiation enteritis. In addition, systemic therapies such as hormonal therapy, nonsteroidal anti-inflammatory drugs (NSAIDs), chemotherapy, and targeted therapies such as tyrosine kinase inhibitors and antiangiogenic drugs can be utilized for medical management [5].

## Conclusion

Overall, DTFs are rare soft tissue tumors. Because DTFs can occur in a wide variety of locations, such as chest wall or abdomen, they can simulate mesothelioma. This case illustrates the use of immunohistochemistry to differentiate similar rare pathologies in order to provide correct treatment and management for patients. Currently, our patient is alive and undergoing active surveillance for both peritoneal mesothelioma and DTF, with no signs of recurrence. In addition, due to the benign nature of the pathology and complete surgical resection, we did not feel that further systemic chemotherapy was appropriate at this time. In the future, suspicious lesions in this patient may be biopsied to accurately differentiate etiology using IHC. It is important to note that while DTFs have initially been diagnosed as malignant peritoneal mesothelioma, there have been no case reports to our knowledge of DTFs in the setting of an additional diagnosis of malignant peritoneal mesothelioma.

## Data Availability

Data sharing is not applicable to this article as no datasets were generated or analyzed.

## Abbreviations

HIPEC: Hyperthermic intraperitoneal chemotherapy
DTF: Desmoid-type fibromatosis
FAP: Familial adenomatous polyposis
PCI: Peritoneal cancer index
